# Food Waste Utilization for Reducing Carbon Footprints towards Sustainable and Cleaner Environment: A Review

**DOI:** 10.3390/ijerph20032318

**Published:** 2023-01-28

**Authors:** Latika Bhatia, Harit Jha, Tanushree Sarkar, Prakash Kumar Sarangi

**Affiliations:** 1 Department of Microbiology & Bioinformatics, Atal Bihari Vajpayee University, Bilaspur 495001, India; 2 Department of Biotechnology, Guru Ghasidas University, Bilaspur 495009, India; 3 College of Agriculture, Central Agricultural University, Imphal 795004, India

**Keywords:** food waste, anaerobic digestion, animal feed, compost, sustainability, biorefinery, biofuel

## Abstract

There is world-wide generation of food waste daily in significant amounts, leading to depletion of natural resources and deteriorating air quality. One-third of global food produced is wasted laterally with the food value chain. Carbon footprint is an efficient way of communicating the issues related to climate change and the necessity of changing behavior. Valorization or utilization of food wastes helps in resolving issues related to environment pollution. Reduction in the carbon footprint throughout the chain of food supply makes the whole process eco-friendly. Prevailing food waste disposal systems focus on their economic and environmental viability and are putting efforts into using food waste as a resource input to agriculture. Effective and advanced waste management systems are adopted to deal with massive waste production so as to fill the gap between the production and management of waste disposal. Food waste biorefineries are a sustainable, eco-friendly, and cost-effective approach for the production of platform chemicals, biofuels, and other bio-based materials. These materials not only provide sustainable resources for producing various chemicals and materials but have the potential to reduce this huge environmental burden significantly. In this regard, technological advancement has occurred in past few years that has proven suitable for tackling this problem.

## 1. Introduction

The world is facing many problems that demand timely attention so that the sustainability of mankind is ensured. The chief factors among these issues are food security, the impact of greenhouse gases emission, depleting reservoirs of fossil fuels, and food waste generation and management [1]. The past few decades have witnessed a rise in population at the global level, which has put pressure on the availability of food, its management, and its disposal. A global rise in the emission of greenhouse gases has deteriorated the climate, which is not only a burning issue is also centrally placed all over the world. The world is exploring novel pathways to solve some of its global issues. The solutions for these global issues are being extensively explored [2]. A continuous rise in human and domesticated livestock populations globally is showing an impact on the use of resources and depicts huge ecological footprints (EFP) [3]. The consequences of humanity’s ever-rising EFP is leading to a hike in climate change, water scarceness and contamination, degradation of soil, and diminishing biodiversity above and below ground. There are several sub-sections of EFP. Carbon footprint (CFP) is one of these sub-sections, along with land footprints, water footprints, nitrogen footprints, biodiversity footprints, power footprints, etc. [4].

Carbon footprint may be defined as “Carbon dioxide in tonnes equivalent to greenhouse gases produced due to anthropogenic activities” [5]. Thus, a carbon footprint is the total greenhouse emissions emanating from a product, activity, or an organization. The CFP is reported as CO_2_ eq by converting methane and nitrogen oxides into CO_2_. It is well-established that the global per capita CFP due to anthropogenic activities is not sustainable and may lead to an increase in global warming above 2 °C [6]. Food waste is one of the important factors contributing to CFP and thereby increasing global temperature. It is thus pertinent to reduce food waste and consequently CFP. The treatment of food waste by different sustainable methods is paramount to create a positive impact on the CFP and the global environment [7].

The past few decades have witnessed the massive growth, industrialization, and modernization of the food and agro-industrial sector, which have revolutionized them in a magnificent way [6]. As an outcome of these developments, there has been a drastic escalation in the productivity and marketing of the food and agro-industrial sector, leading to the generation of agro-industrial food waste. A constant rise in food waste generation is the outcome of continuing economic and population growth in developing Asian countries. Food is a precious commodity that not only rapidly converts to waste but also negatively influences the environment [8]. Food waste is also associated with several other losses. Loss of profit, futile resources, and labor hours are among the important losses that accompany food waste. Social problems are also associated with the generation of FW around the globe. Food waste deprives a huge population of food and is also a threat to the environment [5]. Food waste is comprised of a wide range of residual wastes and post-consumption wastes, with the former being produced at any step of the food production supply chain and the latter generating from domestic or commercial activities. Usually, the nature of food waste is complex, as it is made of various types of palatable substances. Food waste is a rich source of carbon and has a huge energy potential. It is considered as an abundant biomass resource globally. The food industry generates an enormous amount of food waste along with other end-consumers such as home and restaurant environments. Food waste generated from homes and restaurants becomes a prominent part of municipal waste [9]. Food waste is also generated from hotels, food markets, cafeterias, and bakeries. The high consumption rate of food and very low recycle rate of food waste leads to the formation of mountains of food discarded into landfills. In these landfills, the dumped food is burned, thereby producing harmful gases [10]. This practice is highly damaging, as it causes severe human health issues arising due to air pollution and soil leaching. Food wastes generates a significant amount of greenhouse gases. The worldwide carbon footprint (CF) of yearly food waste is approximately 3.3 Gt carbon dioxide equivalent (CO_2_e) [11].

It is the need of the hour to develop strategies to curtail food wastage in order to sustain the economy and to combat the change in climate [12]. The most popular and traditional ways to combat food waste are landfilling, anaerobic digestion, incineration, composting, use in fertilizer supply, and usage as animal feed [13]. In current scenario, different strategies are being researched and adopted for the effective valorization and reutilization of food waste so that environmental and social issues can be eradicated. The most recent few decades have witnessed the involvement of agro-industrial food waste as a biorefinery for the production of commercially feasible products such as biochemical, biofuels, and organic acids. This strategy could successfully replace the conventional methods of disposing food waste in landfills [14].

As the inefficient use of food resources leads to loss of monetary value, it is best to transform them into a product that will be cost-effective and eco-friendly in terms of its carbon footprints. It is necessary to understand the techno-economic challenges of food waste biorefineries in order to assure their sustainable development [2]. Furthermore, it is also associated with the Sustainable Development Goal (SDG) number 12 from the United Nations, i.e., “Ensure sustainable consumption and production patterns”, that launches an aim that “by 2030, the global food waste per capita should be halved at the level of the retail and consumer along with the reduction of food wastes along production and supply chains” [12].

This review work presents a comprehensive outlook on the management strategies adopted to combat with food waste for socioeconomic benefit. Complex nature of agro-industrial food waste and various technologies involved in conversion of these food waste into commercially valuable products are also discussed. This review will give detailed account for the producing various product out of food wastes which are not only sustainable and cost effective, but eco-friendly too in terms of their carbon foot prints. Emphasis is put to address the challenges faced by food waste bio refineries in order to get commercialized and to promote the perception of circular bio economy. 

## 2. Food Waste Generation 

When the final products of numerous food processing industries are not used or recycled for any other purpose, it is defined as food waste (FW). The economic value of these non-product flows of raw materials is less in comparison to cost involved in their collection and recovery for recycle. Hence, they are rejected as waste. European union generates about 89 million tonnes of FW annually. Eighty percent of this total figure is jointly contributed by household sector (42%) and manufacturing sector (38%) [15]. This shows that food waste is produced at every phase of food supply chain. Waste generated during food supply chain is known as food supply chain waste (FSCW). This waste is mostly organic material produced for human consumption, which is lost, rejected, or decomposed at the manufacturing and retail stages. An anticipated 14% of the world’s food gets wasted between harvest and retail [16]. Generation of food waste is more obvious at the retail and consumer stage of food supply chain. About 17% is of food waste is generated at the retail and consumer stage of food supply chain. According to FAO, 50% of the food produced is lost or wasted before and after reaching the consumer, which accounts for more than 1.3 billion tons of food produced per year for human intake. For instance, food waste generated at consumer level in wealthy nations is predictable to be 222 MT, which is very close to Sub-Saharan Africa’s total net food production (230 MT) [17]. The agro-food supply chain is comprised of a wide diversity of production procedures that produce huge amount of diverse waste, particularly organic residues. FSCW includes waste generated from food spoilage or pest degradation [18,19,20].

**Figure 1:**
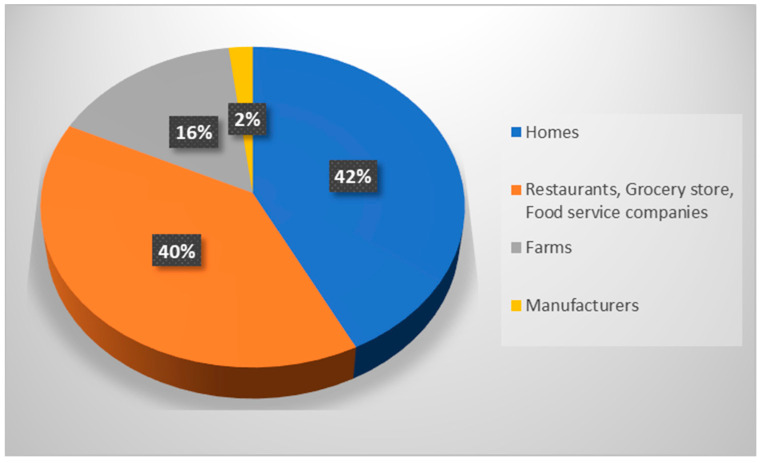
Percentage of food waste generated at various levels of food supply chain in America.

While the global generation of food wastes per year is about 1.4 billion tons, the United States discards nearly 40 million tons worth 80 billion pounds of food per year, which is more than any other country in the world. This food waste in US accounts for 30–40% of the whole US food supply and is equal to 219 pounds of waste per person. It is just like every individual in America is throwing more than 650 average sized apples straight into the dust bin—or relatively straight into landfills, as discarded food mostly ends up there. US landfills are mostly occupied by food wastes that makes up 22% of municipal solid waste (MSW). The percentage share of food waste contributed at various stages of food supply chain in USA is given in Figure 1.

It is estimated that about 30 to 40% of the food produced by farmers globally, is never consumed. At the manufacturing level, more than 10% of food is wasted due to human errors that includes their poor training and lack of standard operating processes. In America, about 30% of food is thrown away by grocery stores and the retail stores of US produces about 16 billion pounds of food waste annually. According to FAO, about 750 billion US dollars is the economic cost of food wasted of agricultural products excluding fish and seafood. The annual value of food wasted worldwide is one trillion dollars. According to the environmental Protection Agaency (EPA), 2.6 MT of food was composted in 2018, which is just 4.1% of wasted food. Globally, the maximum percentage of total food wastage volumes (33%) is contributed by agricultural production. About 54% and 46% of food wastes is the upstream (production, post-harvest handling and storage) and downstream (processing, distribution and consumption) waste volume respectively. These apparently signify a main social, environmental and economic problem [21].

## 3. Technologies for Food Waste Utilization for Low Carbon Footprints 

Environment safety of a country and world is assured when a responsible waste management becomes an integral part of it. Exploring the cost effective and eco-friendly technologies for recycling food wastes helps in the production of valuable products of commercial importance. Modern technological approaches help in resolving the issues of waste management particularly food wastes [22]. Execution of effective methods for recycling of waste are appropriate tasks along with reduction of landfills and separate collection of garbage. Current researchers recommend novel approaches to circulate waste by producing energy and bio-active compounds [23]. Improved methods of Hydrothermal carbonization, dendro liquid energy (DLE), ultra-fast hydrolysis, anaerobic digestion, composting and pretreatment allow to procure energy, biofuels, fertilizers and material for other industries. Smart waste processing becomes feasible with the development of digital technologies [24].

### 3.1. Hydrothermal Carbonization (HTC)

**Figure 2:**
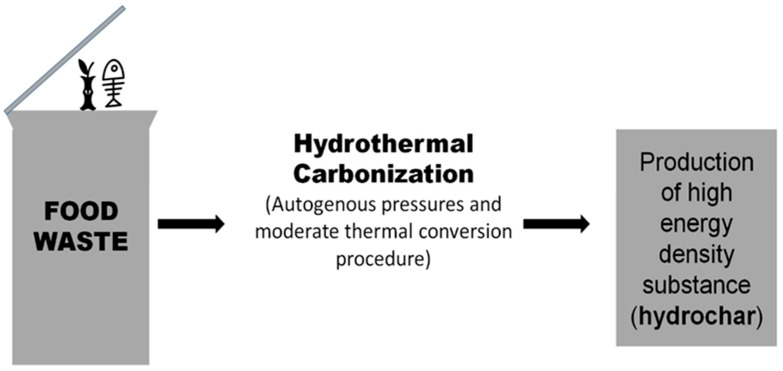
Waste to energy conversion by hydrothermal carbonization.

The thermal conversion method known as hydrothermal carbonization (HTC) has the ability to solve a variety of problems that arise when treating waste food biologically [25]. This technique produces a substance with a significant amount of carbon and high energy density known as hydrochar, which has been said to have a chemical makeup and energy level comparable to lignite coal as depicted in Figure 2. The obtained chars may be conveniently kept and utilised as desired for generating electricity [26]. 

Since HTC uses thermochemistry, heterogeneous wastes will not pose as much of a practical challenge as they do in anaerobic process and decomposition. Additionally, food wastes are better suitable for conversion by HTC than other dry, more widely used thermal transition procedures due to the demand for hydration [27]. Additionally, carbonization generates a product that is rich in energy and is simple to store. HTC is a wet, moderate thermal conversion procedure that takes place under autogenous pressures (180–350 °C) [28].

### 3.2. Dendro Liquid Energy (DLE)

Dendro liquid energy (DLE), an innovative waste-to-energy system that processes organic waste, is undoubtedly the most intriguing promising technology today. If compared to anaerobic digestion and other WTE systems, DLE plants are around four times more productive at harnessing power, since they run at moderate temperatures between 150 °C and 250 °C. Food waste may be used by dendro liquid energy plants to produce clean electricity-generating fuels like carbon monoxide and hydrogen. DLE, on the other hand, keeps costs down because there is no combustion involved in the process, minimizing the need for sophisticated anti-emission equipment [29].

DLE provides a number of key benefits, including effectiveness for power conversion of over 80% and nearly zero greenhouse gas emissions, which means that the syngas and solid waste are free of tar and particle pollution. It is the ideal local option for several communities due to its cheap operational expense. Waste-to-energy engineering will ideally open up more significant exposure to DLE in the near future because, in addition to being an extremely profitable industry, the recurring energy crisis has shown how important a certain level of independence [30]. 

### 3.3. Ultra-Fast Hydrolysis

Food waste when subjected to ultra-fast hydrolysis, it can be used to produce biofertilizers and generate power. After hydrolysis, the segregated solids portion could be easily transformed into biofertilizer, and the liquid can be supplied to microbial fuel cells (MFCs) for instantaneous energy production. With zero-solid discharge, this approach moves the management of food waste forward into extremely rapid concurrent resource and energy recovery. The incorporation of sufficient anodic respiring microbes around the anode might further strengthen or boost the efficiency of MFCs [31]. The versatility of microbes to utilise a variety of wastes as fuels for activities makes it a suitable approach for efficient bioelectricity production from various organic matter. The better comprehend of the bio-electrochemical MFCs, which come in single- and double-chamber formats and with or without proton exchange membranes, differ greatly (PEM). PEMs have other advantageous qualities such strong cation conductance, relatively low resistance, and the capacity to function for prolonged duration of time without interfering with the MFC in addition to being highly specific to protons. PEM is therefore thought to be the membrane separator that is employed extensively in double-chambered MFCs [32].

This technique links microbial activity to electrochemical processes and aids in both the production of electricity and the control of waste generation. Exoelectrogens, which also are commonly referred to as electroactive microbes, possess molecular machinery that enables them to send electrons to an electron acceptor without the requirement of further assistance. The food waste (waste substrate) is oxidised using them as biocatalysts inside the anode compartment, and chemical energy from the oxidation of organic/inorganic molecules is converted into ATP through a series of processes. The “powerhouse of the MFCs” are the microbes that are used to generate electricity from food waste [33].

The electrode material’s surface roughness may boost longevity while also causing corrosion, which lowers MFC efficiency. The suitable materials for MFC electrodes should be robust and long-lasting in both acidic and alkaline environments [34]. An appropriate electrode composition should possess a high current density, be mechanically stable with a broad surface area, and also have favourable electrochemical attributes (electron transfer). Microbes have a special ability to transmit electrons from the cell’s cell outer layer towards to anode and catalyse reduced electron acceptors by taking electrons from the cathode surface, which is a significant element in the operation of MFCs [35]. Biofilm is a thin layer of microbial cell growth formed on the electrodes by microbes connected to MFC. The site where the biofilm forms controls the microbes’ capacity to generate electrons and has a significant effect on the breakdown of the diverse organic substances in MFC. Numerous bacterial species, including *Shewanella*, have been identified as exoelectrogens or electrogenic bacteria. These bacteria serve as catalysts for the digestion of organic waste and the breakdown of carbon-based organic matter as well as the production of power [36].

Microbial fuel cell technique has the chance to be a futuristic, efficient and environment friendly solution to the world’s energy needs and the current energy problem. The current methods for generating electricity are entirely dependent on other natural energy reserves, which are quickly running out owing to overuse. MFC implementation and utilization is moving toward the mainstream as a multi-system for waste management and power restoration at the same time [37].

### 3.4. Anaerobic Digestion 

**Figure 3:**
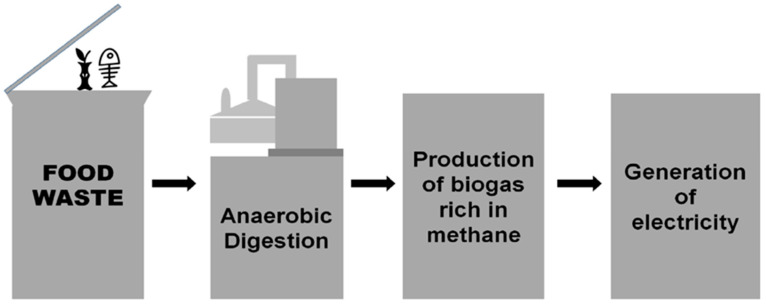
Waste to energy conversion by anaerobic digestion.

Anaerobic digestion is a known and established technique for the production of biogas and is considered ideal for food waste management. This technique has gathered attention in past few decades due to its promotion by various national programmes for the generation of renewable energy. Biodegradable substrates like agricultural or industrial food waste, food industry wastes and municipal solid waste can be transformed into biogas with the help of anaerobic digestion. It is a carbon capture strategy from food waste during its digestion in which hydrogen and carbon dioxide from various sources are converted into methane. Food waste anaerobic digestion (AD) has received considerable attention as a sustainable method of managing food waste with the objective of volume minimization and energy recuperation in the form of bioenergy (Figure 3).

However, the actual proof that is currently available indicates that AD has numerous disadvantages, including a lengthy solid retention period of 15 to 20 days, an immensely complicated procedure configuration that combines hydrolysis, acidogenesis, acetogenesis, and methanogenesis. Ineffective biomethane to electricity conversion and low percentage of food waste reduction are also considered as disadvantages of anaerobic digestion. Further high maintenance cost and safety issues are major concern related to AD [38].

### 3.5. Gasification

Due to the fact that gasification’s end product (syngas) is treated prior to (rather than after) use, it is widely regarded as a significantly superior thermal WTE approach over incineration. In other terms, compared to conventional incinerators, gasification waste-to-energy systems emit significantly lesser pollutants [39]. Municipal garbage is converted into syngas using gasification, which uses it as a substrate instead of fuel. Syngas is a flammable synthetic gas that can be employed for fertilizers, as a substitute for natural gas, and as a source of gasoline. Take into consideration that not every elements are appropriate for gasification, hence the majority of gasification plants typically necessitate screening and pretreatment of municipal garbage [40,41,42].

### 3.6. Pretreatment

Food wastes and agricultural wastes are excellent substrates for the production of second generation bioethanol. Ethanol is metabolically produced by yeast and some bacteria by consuming monomeric sugars. These organisms cannot covert starch or cellulose directly into ethanol. These polymers need to be broken down into their monomeric constituent ie glucose, in order to be utilized for bioethanol production [43]. Various pretreatment techniques help in the conversion of polymers into monomers. Many food wastes like peels of fruits and vegetables are lignocellulosic biomass and are chiefly comprised of cellulose, hemicellulose and lignin. Glucose (hexose) is the main sugar of cellulose liberated upon its degradation, whereas hemicellulose is rich in both pentose (arabinose and xylose) and hexose sugars (glucose, galactose and mannose) [44]. Pretreatment of lignocellulosic material with physiochemical, chemical or enzymatic methods helps in breaking the association between cellulose, hemicellulose and lignin, thereby liberating these hexose and pentose sugars for further fermentation by suitable organism. Researches are now focusing on utilizing these natural sources of sugars for biofuel production [45]. 

Pretreatments are commonly used to enhance the carbohydrate saccharification of organic substrates. Kitchen waste are nutrient rich media that efficiently supports the growth of ethanologenic microorganism to produce high ethanol yields [46]. Hence pretreatment is not needed for kitchen waste prior to enzymatic hydrolysis to attain high glucose levels. About 0.3 and 0.4 g ethanol is produced per gramme total solids of carbohydrate-rich food wastes. Waste fruits are excellent substrates for might bioethanol production. Banana waste or rotten banana, fruit peels, and low-quality fruits, for instance, have been extensively explored as substrates for bioethanol production [47].

## 4. Low Carbon Products for Sustainable and Cleaner Environment 

**Figure 4:**
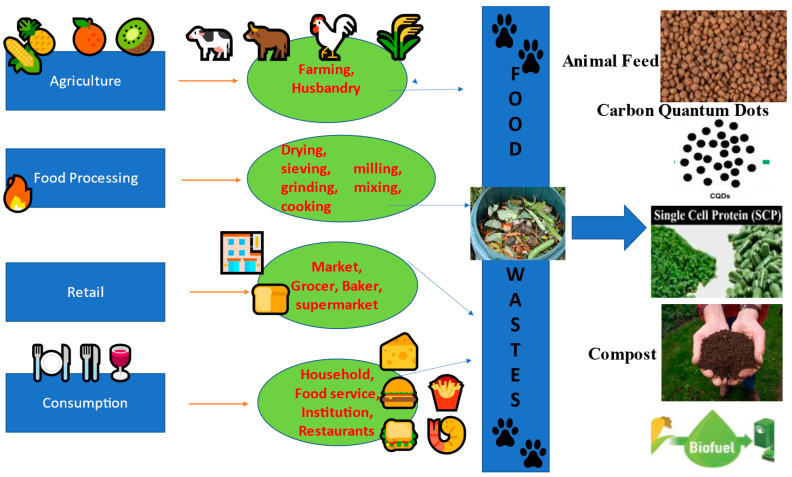
Commercial utilization of food wastes generated from various places and processes.

Landfills are conventional method of disposal of food waste generated from residential, commercial, industrial and institutional sources. Industrial waste as well as the waste generated from food industry are the active participants in promoting pollution and causes a huge negative impact on environment. Many technologies are being investigated that would generate fuels, power, or products from food waste, thereby valorizing and nurturing the growth of a circular economy for food waste. Substantial resources could be generated when food wastes are disposed to these technologies. Waste gets minimized when food industry waste is converted to fuel, feed or food. These conversions not only curtail the cost of food products, but also confirms environmental advantages and huge value of money. Fuels like biodiesel, ethanol, methanol, hydrogen gas and methane gas can be manufactured by utilizing food waste [14]. List of products that can be produced out of food waste is mentioned in this section and are depicted in Figure 4.

### 4.1. Animal Feed 

Animal feed can become an ultimate option to combat with food waste. Waste generated from poultry slaughterhouse is rich source of essential amino acid and can efficiently nourish ruminants. This practice is an efficient strategy to prevent environmental pollution, as this product is clean. In present times, the poultry farming has to abide the certified organic standards and these methods of alternative feeding helps to utilize the innovative and alternative protein feeds thereby contributing towards an ecological modes of poultry production. This alternative approach of converting food waste to animal feeds as it allows the integration of nutrient strategies into overall production system, thereby making the animal production system cost effective and eco-friendly [48].

Research was also conducted to evaluate the potential of sugar pulp and potato peels with or without the addition of enzymes for the diets of broiler chicken. As these food wastes are rich in non-starch polysaccharides like cellulose, hemicellulose, xylose, and lignin, and that the poultry do not possess the enzymes to degrade these polysaccharides. Table 1 shows the various food wastes that are being researched to be used as animal feed.

In countries like South Korea, Taiwan and Japan, local laws executed to support the usage of food waste to feed livestock compiles, 72.1, 33 and 81%, respectively, of entire food waste generation [51]. There are some concerns associated when the food waste is to be redirected to animal feed as an option. Policies refrains from feeding ruminant animals with mammalian proteins and these policies must be strictly followed in order to safeguard the animals and, ultimately, human consumers (U.S. Food and Drug Administration (FDA), 2018). Mostly the moisture content of food waste is very high (>80%) and needs to reduced to about 10% to be used as animal feed [52]. Processing techniques helps in the mitigation of these risk factors to use food waste as animal feed. 

### 4.2. Preparation of Carbon Quantum Dots (CQDs)

Carbon quantum dots (CQDs) are popular for their unique physical and optical properties and thus has gathered significant attention too. Different fields apply various kinds of sources of carbon, but when it comes to food industry, CQDs used needs to be safe. The best way of preparing CQDs for food industry is the use of natural materials without the involvement of chemicals [53]. Nevertheless, food wastes are often overlooked. When used in an efficient way, the food waste resolves many issues related with global economy environmental pollution. When food waste is used as a source of carbon, it not only enables food safety detection but also enhances the value of by-product. Scientists [11] researched on progress of employment of food waste as carbon precursor and its usages in food safety detection. Efforts are done in preparation, characterization and applications of CQDs from various types of food wastes [13]. Detection of heavy metal ions and food additives is necessary to ensure food quality and safety. Presently, food waste as a source of carbon could be classified as the by-products of plant, animal food and food processing. Food wastes serves as a source of carbon precursor for the preparation of CQDs which are then employed for detection of food additives and heavy metal ions. Hence, food waste has potential for the preparation of CQDs and be used to detect food safety [11].

### 4.3. Protein Production

All living organisms needs food for their growth and development. Protein is an important constituent of food, that are considered as a building block of life forms. Significant functions of cells, tissues, organs, and systems are sometimes dependent on protein. A food system comprises lipids, carbohydrates and proteins as three chief chemical components. Massive growth in human population has created a challenge in terms of availability of food. The concern for feeding this huge population nutritious food is the concern of many [54]. Other parallel concerns are the adequate supply of proteins of appropriate quality to maintain health. An alternative source to protein are being explored by researchers in order to suffice all these concerns. About 190 million tons of wastes is produced by the agro-food industry in the form of animal and plant proteins. Plant waste like pomace, oilcakes, sunflower nuts, soybean, wheat, rice, pea, seeds, peel are rich source of proteins and can suffice the protein requirement of daily appetite. Similarly, animal proteins comprises of whey (milk waste) protein, fish bones, collagen, skin (fish wastes), liver, skin, poultry carcasses, feathers etc. All these food wastes are complete proteins as they have balanced combination of all amino acids which that are indispensable for an organism’s diet [55].

Several recovery techniques help in the valorization of protein waste based on its availability and quantity. Valorization of nutrients rich food wastes helps in meeting the hike in demand of the protein-rich foods and the crunch to avail it [56]. This is an eco-friendly approach to deal with food waste, thereby reducing its carbon footprints. It is also an economic approach as the product is of commercial significance and has been generated out of waste. This dual advantage will meet the necessity of protein demand and the concept of zero waste could be materialized [57].

Single cell proteins (SCP) also known as microbial protein are procured by harvesting and drying the microbial biomass [58]. Submerged fermentation and solid-state fermentation are commonly employed for producing SCP [59]. SCPs are excellent source of essential amino acids and can be produced in short time and has potential to substitute costly sources of protein [60]. Microorganisms can be grown on wide range of substrates/food-wastes and can generate products of industrial engrossment. These products can be proteins and enzymes. Researchers round the globe are working on the recovery and utilization of food industry-based wastes in order to meet the world’s nutritional demand [58]. Table 2 depicts various microorganisms, food waste and growth parameters for production of protein and other metabolites.

### 4.4. Composting

Composting is a biologically mediated process, in which organic material is degraded by microorganisms. It is an aerobic process leading to the formation of biologically stabilized material. It is an efficient approach to reduce carbon footprints of food waste [64]. Along with oxygen, there is also a need of carbon, nitrogen and water in suitable proportions that enables the biological activity required to degrade the organic material. Stability of environmental factors, type and content of food waste, pH and temperature is necessary for the stable degradation process [65]. The process of composting takes over several months and is completed in three phases in sequence. Temperature of the compost and the domination of microorganisms existing varies in all these phases. Bacteria, fungi, and actinobacteria (actinomycetes) are involved in the process of composting [6]. The biological decomposition of food waste gets promoted if some probiotic microbes are added during the composting process. Composting is an efficient way of valorizing food waste and to produce organic fertilizer, cultivation soil, or soil conditioner. Storing, handling and transportation of biofertilizer produced through composting is easy and that makes composting a popular practice for recycling food wastes [66]. In Taiwan, composting plants produces organic fertilizers that applied in the cultivation of fruits (e.g., pineapple), vegetables (e.g., cabbage) and special crops (e.g., tea). About 1000 metric tons of compost products are produced by official food-waste-composting plant, situated in Taiwan city. These compost products were delivered by the farmer associations at a price of about NT $5/kg (US$ 0.17/kg). The products of composting have insignificant carbon foot prints in comparison to food wastes. Bio-fertilizers produced as a result of composting further curtails the carbon footprint by curtailing the requirement for fossil fuel and chemical fertilizers [67,68]. 

### 4.5. Food Waste Biorefinery

Food waste biorefinery is assuring technology that helps in the valorization of waste thereby minimizing the negative impact on environment and effective application of resources. The products generated from waste via biorefinery helps in minimizing the dependency on fossil fuel and making it easy to switch towards circular economy [68]. Food waste biorefinery allows the conversion of wide ranges of food wastes into bio-based materials, biofuels, platform chemicals etc. For the effective valorization of the food waste, it is necessary to know its composition and interaction of its components. The efficient biorefinery process is selected on the basis of final products desi4red. Applying the concepts of biorefinery helps in the production of broad range of products like biofuels, protein, enzymes, animal feed, flavors and colorants, organic acids, bio-fertilizers, bioplastics simultaneously and sequentially from food wastes [69]. Examples of economically valuable products of food waste biorefineries are given below.

#### 4.5.1. Biofuels

The demand for energy has increased globally and the availability of fossil fuel is limited. Added to this, there has been a negative effect on climate due to the emanation of greenhouse gases from the burning of fossil fuels. These are the alarming signals to explore unconventional and sustainable energy sources [70]. Biomass energy is becoming popular these days as it is clean and sustainable form of energy. It can potentially solve the problems of waste management and energy security. Growth in industrialization and agricultural sector has witnessed the accumulation of huge biowastes like forest residues, food waste, agriculture crop residues, wood processing residues industrial waste and municipal solid waste (MSW) [71]. Biomass from these biowastes have also simultaneously emerged prospective feedstocks for bioenergy production. Production and consumption of biofuel is becoming popular as it will be a novel approach for a sustainable future. Biofuels are sustainable source of energy as they not only curtail the emission of green-house gases but also augments the usage of waste material that are environmental nuisance creating pollution [72]. Few examples of the biofuels that can be generated out of food wastes are given below.

##### Biodiesel

Biodiesel is a biofuel that is an option to conventional diesel fuel. Current fuel engines needs no significant alteration to switch to biodiesel [72]. Production of biodiesel will become a costly, if refined oils are used for its production. Moreover, the controversies are generated on the utilization of food material for generating biofuels. Hence, strategies have now shifted on using the food waste for doing the same. This is a cost effective and an eco-friendly approach, as it utilizes the food waste solving the problem of its disposition and producing a fuel whose carbon footprints are insignificant. Recently, many food wastes are researched upon to generate biodiesel. For example, Yang et al. [73] utilized the waste generated from the production of instant noodle as a feedstock for production of bioethanol and biodiesel. The researchers treated the food waste and were able to alter the food waste to bioethanol and biodiesel at a conversion of 98.5 and 95.4%, respectively. These researchers found that these conversion rates are significant and could potentially support manufacturing scale using waste of instant noodle as a viable raw material for energy production. Likewise, Karmee et al. [74] explored that waste cooking oil could also be an appropriate material for generation of biodiesel. These studies emphasize the use of food waste in a coming time as a biodiesel feedstock. There are also many other ways of generating energy from food wastes.

##### Biohydrogen

Hydrogen is regarded as prospective source of alternative energy, as it is competent, clean and renewable nature. These features have gathered more attentions for its cost-effective production in current time [75]. Hydrogen is a privileged and promising future fuel as its energy content is high (120.7 MJ/kg) and its carbon emission is zero. Space shuttles employ liquid hydrogen as a fuel. As the flammability of hydrogen is high, this property of hydrogen makes its storage a difficult task and a research area of exploration. Presently, the transformation of fossil fuels suffices the prerequisite of hydrogen for many processes. Hydrogen production through these routes has huge greenhouse gas footprints [76]. On the other hand, hydrogen production techniques through biological routes are not only sustainable but carbon neutral too. Agro crops, agro-residues, lignocellulosic material, food processing waste, water plants and algae, and municipal waste matters are examples of some biomass employed for hydrogen production [77]. Biohydrogen (H_2_), is the chief end products of degradation of organic polymer (food waste) along with methane (CH_4_), and bioethanol (CH_4_CH_2_OH). The complete valorization of inedible and discarded part of date fruit (by-products) of date led to the production of 292 mL H_2_/g vs. and 235 mL CH_4_/g vs. [78]. Electrofermentation of food wastes produces a significant amount of volatile fatty acids. These volatile fatty acids yield biohydrogen. Sravan et al. [79] reported that electro-fermentation of food waste yielded 4595 mg/L of volatile fatty acids, which generated up to 26% biohydrogen. Utilization of waste biomass of date is an illustration of bioenergy production by an effective conversion of biomass using biorefinery approaches. Similarly, the food waste collected from restaurants can potentially produce biohydrogen and CH_4_. Approximately 0.61 L/g vs. of biohydrogen and 0.42 L/g vs. of CH_4_ were produced when carbohydrate rich food waste gathered from restaurants were sequentially hydrolysed in acidified leach bed reactors and methanogenic reactors [80].

##### Biogas Production

Biogas (methane) can be produced by anaerobic digestion of food waste. When carried out under optimum temperature and other parameters, the yield of biogas is maximum. The gas formed subsequent to anaerobic digestion is cleaned and dewatered for its further utilization through a biogas-based fuel generation system. The slurry formed in the process as a by-product is of great importance, as it an excellent organic manure for farming and maintains the soil fertility of the soil. Thus, the slurry is a value added by product of methanogenesis process. A simple, easy to operate and economically feasible Organic Processing Digester (OPD) system was fabricated by Singh et al. [6] that can yield biogas in an eco-friendly manner. In this research, the researchers collected the food waste from selected sites of Dhanbad district of India, which were then exposed to anaerobic digestion in Organic Processing Digester. About 43%, 33% and 24% methane was yielded by this process in summer, rainy and winter season respectively. The study proved that the food wastes are extremely attractive substrate for anaerobic digesters and are potential source of sustainable renewable energy due to their high biodegradability and significant methane yield [6]. This study proves that food waste/municipal solid waste (MSW) can be potentially employed to generate energy and are the sustainable approaches divert these wastes from landfilling, thereby reducing the emission of CO_2_ and CH_4_ to the atmosphere. During the anaerobic digestion of waste obtained from the slaughter potentially generate 324,842 Nm^3/^year of biogas (189,383 Nm^3^/year CH_4_). This biogas can be converted into thermal energy (1886 MWh) or electrical energy (660 MWh), thereby preventing the emission of 557 t CO_2_ equivalents by substituting the usage of fossil fuels. Simultaneous production of organic biofertilizers, along with methanogenesis provides dual advantage. It saves the energy used for production of chemical fertilizers and also the dependency on them [81]. 

##### Bioethanol

Biofuels are considered as a clean fuel. They are low carbon fuels that lowers the vehicle emissions. Reduction in oil consumption and emissions of carbon di oxides is an outcome of using biofuels. Many industries are now focusing on the use of biofuels, chief among them are aviation, marine transport, and heavy freight. Ethanol as a biofuel has an efficient combustion property attributed to its energy content having low per volumetric unit [82]. Moreover, engines can operate at higher compression ratio when ethanol is used as fuel. Octane ratings are excellent in ethanol. Total replacement of conventional fuel with bioethanol is rather difficult in most of the regions and hence it is easier to blend gasoline with 10% ethanol. Vehicle designing are now upgrading themselves to meet these new changes which would enhance their efficiency and would permit blending at mid-level of 20–40% [83].

There is a copious production of agro residue both in terms of quantity and quality globally. Being the repertoires of variety of sugars, these agro-residues can serve as a starting material for various biofuel production. These types of biofuels that are generated from crop residues of forest residues are known as second generation biofuels. A group of researchers have produced ethanol from peels of banana, Citrus sinensis var mosambi, Ananas cosmosus and Litchi chinensis [84,85,86]. Other important carbohydrate sources for producing bioethanol are sugarcane bagasse, rice husk, rice straw, Corn stover. Production of biofuels from these lignocellulosic biomasses is a cost effective, eco-friendly, renewable and sustainable approach [87].

#### 4.5.2. Bio-Based Enzymes

Fish wastes are an excellent source for obtaining the enzymes like protease and esterase. These enzymes are of potential importance in industrial and medical industries. Mango waste can be used as a substrate for production of amylase with high enzyme activity (29.23 mg/mL) using the bacteria *Bacillus* sp. F-11. Similarly, protease enzyme (101.98 U/mL) was obtained from shrimp waste with the help of Haloferaxlucentensis GUBF-2 MG076078. Under the optimum parameters (pH 6, NaCl 30% and temperature 42 °C), lipase (5.83 U/mL) could be produced from coconut oil cake. By-product of sugar beet yields 55.15% protein by using the method isoelectric-ammonium sulfate precipitation. It can be said that the food wastes are potentially applicable for extraction and isolation of vital enzymes and proteins from them [88].

#### 4.5.3. Bio-Based Compounds and Materials

Bioactive compounds are amongst the high commercially valuable products and can be potentially obtained from a broad spectrum of plant-based resources. Nevertheless, the production of bioactive compounds from food waste, particularly plant-based food waste, has gathered enormous attention in recent years. This makes the food waste economically significant [89]. Phenolic compounds are well known for their antibacterial, antiviral, anti-carcinogenic, antioxidants and anti-inflammatory activities. These properties make phenolic compounds eligible to used in medical, pharmaceutical and food industries. Conventional or non-conventional techniques helps in the extraction of these compounds from food wastes. Orange peel (fruit wastes) are rich source of pectin and essential oils. Using microwave irradiation these compounds can be sequentially extracted from this fruit waste. Spent coffee waste is a source of polyphenols (1–1.5%), that can be extracted from it using aqueous ethanol (20%) with microwave irradiation for 40 s at 80 W. About 399 mg GAE/g equivalent of polyphenols was effectively extracted using this technique [23]. There are no standard protocols for the extracting bioactive compounds. This is because of vast diversity of food wastes, chemistry and composition of the wastes, chemistry of the bioactive compounds and the extraction conditions and/or parameters. Hence, it becomes very important to develop more efficient and effective methods of extraction of bioactive compounds from particular food waste. This will be the vital step for successful contribution toward the circular bioeconomy. 

**Figure 5:**
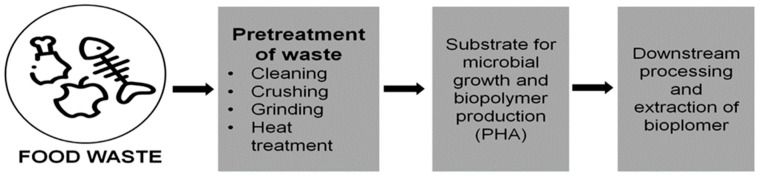
Production of bioplastic from food waste.

The excellent water vapour barrier, stronger fat/odor barrier, and good oxygen barrier (than both non-biodegradable polypropylene (PP) and polyethylene terephthalate) are known as PHA’s outstanding characteristics. PHA’s improved physico-chemical qualities—for instance, in comparison to PP—have encouraged its use in a variety of industries, particularly fine chemicals, pharmaceutical industries, and food packaging material. Numerous investigations have been carried out in an attempt to discover low-cost feedstocks that can be used on a larger scale than standard raw materials [90]. Agro-industrial waste (such as lignocellulosic waste from the food industry and forestry) has received a great deal of attention among many of the multiple substitute feedstocks for PHA production because food waste offers a tactical way to reduce the ultimate manufacturing cost. Polymers can be synthesised from carbon-neutral substances in the fabrication of bioplastics made from food waste, which is an environmentally friendly and regenerative process (Figure 5). 

## 5. Future Prospective

For future sustainability, food waste can be a great hope among the available renewable energy sources. Municipal solid waste (MSW), which is regarded as a clean energy resource and comprises a substantial amount of food waste, is a resource for biomass [91]. Additionally, food waste products may be converted into usable power sources as biohydrogen (hydrogen), biogas, bioalcohols, and so on, with the right waste-to-energy (WTE) methods. WTE’s main objectives include reducing carbon footprint and developing fossil fuel substitutes [92]. 

Utilizing Household food waste (HFW) as a primary resource is a significant problem because it is challenging to recover produced HFW from various locations and to manage it after recovery. Microbes may find HFW to be an obvious target due to humidity and total carbohydrate, which could cause significant deterioration [93]. The variability of HFW, which is significantly impacted by the location from which the wastes are obtained, presents additional difficulty. The content of the HFW can also be influenced by dietary practises and the time of year it is collected. Typically, a sizeable amount of the wastes are made up of fruits and vegetables [94]. The correct training of the general public is a crucial problem that must be resolved in order to attain low contaminant prevalence throughout source segregation of HFW.

The majority of recent studies has emphasized on so-called second-generation bioenergy, which employ wastes or by-products as raw resources as opposed to first-generation bioenergy, which used sugars and starch. In this approach, the public’s growing concerns about using food sources to make bioenergy can be allayed. After all, using grains or sugar to make biofuels has caused their prices to skyrocket globally, which has caused major issues for the developing nations. All of these worries sparked a surge in study into using residues and wastes at minimal cost as raw materials. Utilizing organic wastes, especially home food wastes, as a separate and substitute resource of raw materials for the generation of biofuels is possible (HFW) [95].

Usage of source-separated HFW is used at high dry substrate levels for ethanol fermentation in order to generate maximum ethanol synthesis. When HFW is used at high dry substrate levels, the resulting mash is extremely viscous, making solid-state culture the only viable option. This has several drawbacks, notably challenges with operation scaling-up and ethanol collection [96]. To get over this problem, commercial cellulases solutions were used in an enzymatic saccharification technique before processing. The high solid percentage substrate’s viscosity was quickly minimized throughout this method, allowing submerged culture. To reduce the rate of soluble sugar breakdown, no pre-processing was used before enzymatic saccharification. The residual solids (residue) were then subjected to a further processing and fermentation in order to enhance the ethanol production [97].

The majority of advanced countries, including certain European nations and Japan, have adopted cutting-edge waste treatment technology aimed at minimizing final waste dumping while generating electricity and/or thermal energy, hence lowering power usage [98]. Energy recovery or WTE methods including anaerobic digestion (AD), incineration, refuse-derived fuel, and pyrolysis are currently being used to create a variety of significant outputs like power, heat, fuels, hydrocarbons, and biofertilizer [99]. Although the majority of WTE techniques have the potential to produce electricity, WTE facilities are now searching for integrated power and heat production. The opportunity exists for new cutting-edge facilities to convert waste into fuel source and a biogas replacement [100]. 

Food waste by-products in farming are advantageous and might reduce farmer’s usage of synthetic chemical additives, in some instances completely replacing such chemicals. Consequently, products would cost less. By allowing cultivators to use food waste by-products for agricultural uses, industries aid society’s shift towards a more environmentally friendly model of consumption [101]. For food waste management, the production of bioplastics like polyhydroxyalkanoates (PHA) is suitable. The fact that food waste is landfilled and produces unfavourable outcomes like greenhouse gas (GHG) pollution and contaminated groundwater is one justification for the same. Polymers can be synthesised from carbon-neutral substances in the fabrication of bioplastics made from food waste, which is an environmentally friendly and regenerative process [102]. Under commercial circumstances, some bioplastics are biodegradable and recyclable. Biopolymers can be disposed breakdown by 90% in a few months, but they can also be composted in industrial setups [103]. 

One of the main components influencing the business for biodegradable polymers is PHA. PHA is a significant category of polymers that has previously been in investigation but is now ready to fully participate in the market, where production capacity is expected to treble in the upcoming few years. Its degradation rate and rubber-like qualities have revealed that it has tremendous opportunities as a replacement for conventional plastics [104,105]. The 2030 Agenda for Sustainable Development—specifically SDG 12, target 12.3—demands for per-capita universal food waste at the retail and consumer levels to half and curtailing the loss of food along production and supply chains. As only eight years are left to attain this target, there is an urgency to scale up action to lessen the food waste [106,107,108,109]. Reducing the food waste provides an opportunity for instantaneous climate benefits and making our food systems overall sustainable—a obligatory revolution to safeguard this planet and to assure sustainable nutritional outcomes [7,90,110]. Food waste utilization not only aid towards sustainable environment but also provide nutritional and food & energy security [111,112,113,114,115,116,117,118,119,120,121,122,123,124]. 

## 6. Conclusions

Reducing carbon footprints is an important issue of global concern. Revolution in agro-industrial sectors have revolutionized the production of food material and sequentially the food waste. These food waste has a huge negative impact on environment as well as for society, as it is responsible for increasing the carbon footprints and the depletion of important resources that can serve the mankind. These footprints need to be managed and reduced which is possible only with the effective management of food waste. Proper management of food waste can efficiently curtail the emission of can greenhouse gases and dependency on fossil fuels along with the use of nonfood crops. As a substitute of being landfilled, food waste can be directed towards diverse food waste treatment plants. The food wastes are converted into a valuable products that are not only eco-friendly but also highlights a good business sense on a number of enumeration. The field of waste management is now technologically advanced and this has proven to be a significant asset for dealing with food waste. Some alternative technologies are need to be explored in order to overcome the problem associated with effective utilization of wide range of food wastes in an economic and eco-friendly manner. The incorporation of food waste into the bioeconomy is an anticipated task both for the present and future.

## Figures and Tables

**Table 1:** Various food wastes used as animal feed.

For Rearing	Food Waste Used	Characteristic Studied	Observations	Conclusion Drawn	Remarks	Reference
Livestock	Cauliflower and Romanesco waste (e.g., leaves, stems, and sprouts)	Ruminal fermentation and intestinal digestibility	All fractions of food waste were highly degradable and resulted in high digestibility for a protein source.	Up to 24% of dried cauliflower in concentrate can be included in feed for dairy sheep without negative ruminal fermentation effects.	Use of food waste in ruminant nutrition is promising	[49]
Polyculture of low trophic level fish (grass carp, bighead, and mud carp).	Diet A fruits and vegetables—10%, cereal—53%, bone meal—8%, fish meal—10%, corn starch—15%, and other food waste—4%	Presence of plankton, water quality, and fish growth.	Density of planktons was the highest in the Diet A body of water, but not significantly different.	The water quality did not appear to be significantly impacted by the various feeds.	Diet A was devoid of meat waste and was 53% cereal-based	[50]
Polyculture of low trophic level fish (grass carp, bighead, and mud carp).	Diet B 10% fruits and vegetables, 25% meat products, 28% cereal, 8% bone meal, 10% fish meal, 15% corn starch and 4% other food waste.	Presence of plankton, water quality, and fish growth.	This proved to be a better formulation in terms of the complete performance on fish growth factors.	The improved diversification in generation of feedstuff could permit more food waste to be employed for many fishery species.	Diet B was 25% meat and 28% cereal.	[50]

**Table 2:** Various microorganisms, food waste and growth parameters for producing protein and other metabolites.

Microorganism	Food Waste (Substrate)	Growth Parameters	Product	Reference
*K. marxianus* IMB3 (thermotolerant),	Whey, orange and potato residues, molasses, brewer’s	30 °C and pH 7	Aroma compound pinene, protein, and lipid.	[61]
Kefir culture		30 °C and pH 5.5	Aroma compound pinene 4 kg ton^−1^ of the food waste	
*S. cerevisiae* AXAZ-1 (alcohol resistant and psychrotolerant)		30 °C and pH 5.5	38.5% protein	
*Candida utilis* and *Rhizopus oligosporus*	Wheat bran	30 °C and 48 h	Protein yield 41.02%	[62]
*Aphanothece microscopca* Nageli	Rice effluent	30 °C and 72 h	Significant yield of SCP and high ratio of PUFA (mainly gamma linolenic acid)	[58]
*Saccharomyces cerevisiae* BY4743	Yam peel	96 h	Protein having threonine, lysine, valine, and leucine (essential amino acids)	[63]

## Data Availability

Not applicable.
